# Bisphosphonate-Related Osteonecrosis of the Jaw Mimicking Bone Metastasis

**DOI:** 10.1155/2014/281812

**Published:** 2014-01-30

**Authors:** Geetika Bhatt, Aashish Bhatt, Anthony E. Dragun, Xiao-Feng Li, A. Cahid Civelek

**Affiliations:** ^1^Department of Internal Medicine, University of Louisville, 530 S. Jackson Street, Louisville, KY 40202, USA; ^2^Department of Radiation Oncology, The Ohio State University, 300 W 10th Avenue, Columbus, OH 43210, USA; ^3^Department of Radiation Oncology, James Graham Brown Cancer Center, University of Louisville, 530 S. Jackson Street, Louisville, KY 40202, USA; ^4^Division of Nuclear Medicine, Department of Radiology, University of Louisville, 530 South Jackson Street, Suite C07, Louisville, KY 40202, USA

## Abstract

Osteonecrosis of the jaw is usually a potential complication of bisphosphonate therapy. In a cancer patient, this disease entity can be misdiagnosed as a metastatic lesion. Our aim is to make clinicians aware of bisphosphonate associated osteonecrosis of the jaw to prevent misdiagnosis and initiate proper treatment at the earliest. We present the case of a breast cancer patient with multiple bony metastases and a jaw lesion presumed to be metastases. After no response to palliative radiation, repeat radiological imaging studies revealed osteonecrosis of the jaw. Correlating a patient's clinical information with findings on diagnostic imaging studies, such as SPECT bone and CT scans, can help identify this potential complication of bisphosphonate treatment. Early diagnosis helps minimize unnecessary biopsies and allows for the proper treatment to be instituted.

## 1. Introduction

Bisphosphonates are now part of the standard treatment for bone metastases and hypercalcemia of malignancy; their use decreases the incidence of skeletal related events and improves the quality of life [1]. Although the incidence of osteonecrosis of the jaw (ONJ) in the general population is unknown, among cancer patients with metastatic bone disease treated with bisphosphonates it is as high as 10% [2, 3]. The clinical and radiological presentation of bisphosphonate- related osteonecrosis of the jaw (BRONJ) could be mistaken for a bone metastasis [4]. An understanding of the risk factors, preventative measures, early diagnosis, and treatment of BRONJ are thus necessary.

## 2. Case

**Figure 1:**
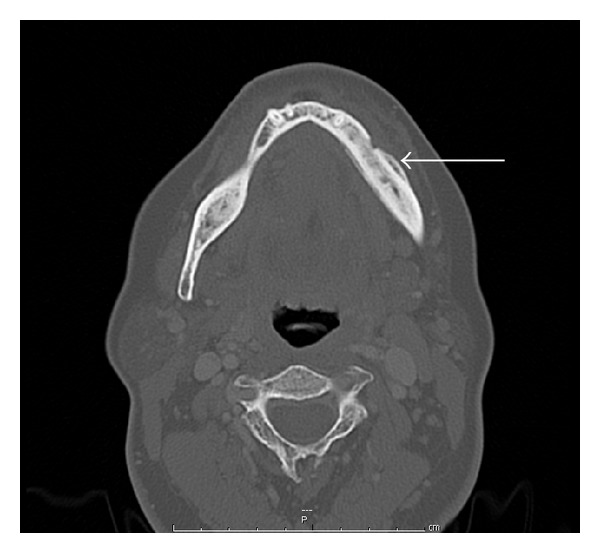
CT scan of the mandible. The CT scan demonstrates thickening of the mandibular body (arrow) with adjacent tissue stranding. This was thought to represent metastatic disease.

**Figure 2:**
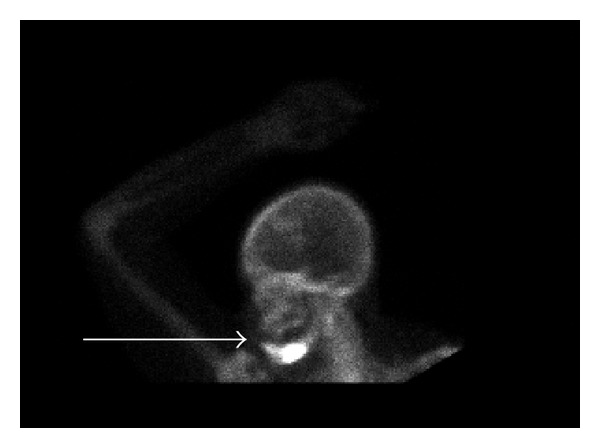
Technetium 99m Bone scintigraphy. Bone scan showing increased radioactive uptake in the body of the mandible (arrow) thought to be metastatic disease.

A 61-year-old African American female was diagnosed with left breast cancer with multiple bone and liver metastases in April 2009. She was treated with chemotherapy and intravenous bisphosphonate therapy with zoledronic acid. A follow-up bone scan redemonstrated the multiple bone metastases, now with a new focus of increased radiotracer activity in the left mandibular angle. Radiological interpretation suggested that this focus most likely represented either severe dental disease or a metastatic focus. Few months later, the patient developed severe pain in her mandible, for which dental evaluation led to a tooth extraction. However, following the tooth extraction, the patient's jaw pain progressed with worsened swelling of the left lower jaw. CT scan of the mandible (Figure 1) demonstrated a mandibular body bony lesion on the left with adjacent soft tissue stranding, again interpreted to be concerning for a metastatic bone lesion. Bone scintigram (Figure 2) demonstrated expansion of the mandibular lesion and slight progression of the multiple other skeletal metastases.

A referral was made to radiation oncology due to worsening jaw pain unresponsive to narcotic medications and imaging findings suggestive of a metastasis. Without exposed bone on physical exam, it was felt that she would benefit from palliative radiotherapy (RT) for her presumed mandibular metastasis. She underwent RT to her jaw lesion to a total dose of 2000 cGy in 5 fractions of 400 cGy each using a 3D-conformal technique. However, after the RT, the patient's jaw pain continued to worsen. Despite negative blood and bone cultures, she received courses of cephalexin and clindamycin, for a presumed jaw infection with no improvement.

**Figure 3:**
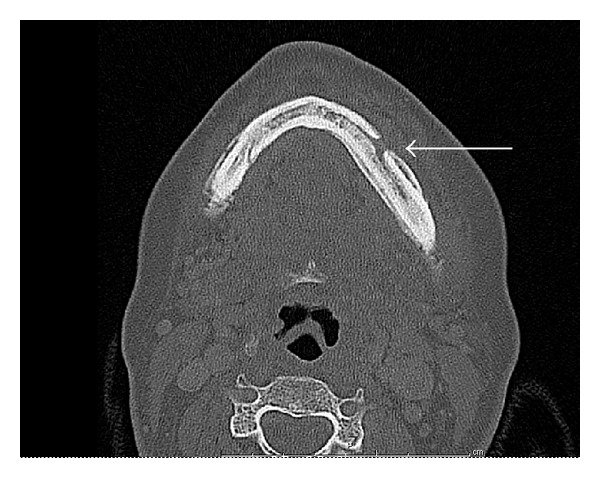
Repeat CT scan of the mandible. Repeat CT scan of the mandible showed changes along the external and internal surface of the mandible (arrow) with production of new bone along the body extending to the mandibular angle thought to be secondary to osteonecrosis from the radiation therapy.

**Figure 4:**
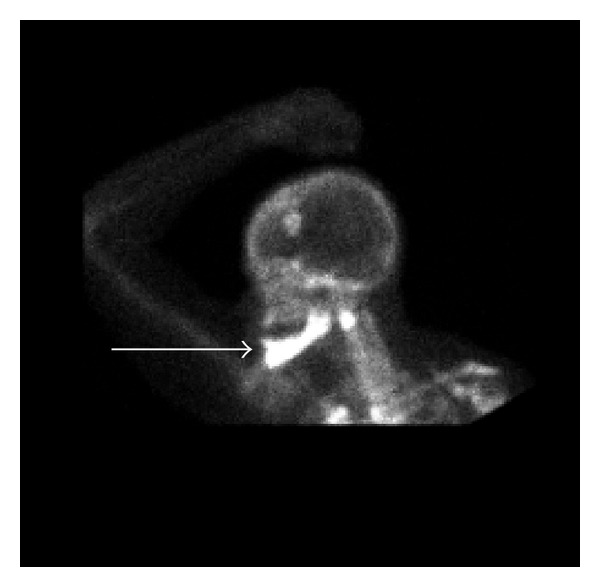
Repeat Technetium 99m bone scintigraphy. Progression of diffuse intense bony radioactivity involving most of the mandible, most likely related to diphosphonate induced mandibular osteonecrosis.

Since the presentation was atypical and there was no therapeutic response, a bone biopsy of the mandibular lesion was obtained but revealed no evidence of metastatic disease. In light of the negative pathology and lack of clinical response, the diagnosis was now felt to be most consistent with BRONJ. Repeat CT (Figure 3) and bone scan (Figure 4) findings were also thought to be consistent with osteonecrosis of the jaw.

## 3. Discussion 

Bisphosphonates bind to osteoclasts and inhibit their ability to resorb bone, causing decreased bone turnover and thus decreasing loss of structural integrity of the bone [5]. According to a review of 368 cases, the patients at the highest risk of BRONJ were patients with multiple myeloma and patients with metastatic carcinoma to the skeleton who were receiving intravenous, nitrogen-containing bisphosphonates (primarily pamidronate or zoledronic acid). Other risk factors for BRONJ include higher cumulative doses of bisphosphonates, history of prior trauma, dental surgery, or dental infection and intravenous administration of bisphosphonates [6].

When BRONJ is present, two-thirds of all patients experience pain, likely secondary to suppuration, infection, sinus tract formation, or exposed bone irritating the adjacent mucosa [6, 7]. Mandible is more commonly affected than the maxilla (~2 : 1 ratio) [6]. Bone exposure is characteristic and is usually present for 8 weeks; however, it may not be seen in the early stages of disease in up to 45% cases [8]. A biopsy may be performed with a high clinical suspicion of metastasis. Caution needs to be exercised as patients suspected of having BRONJ already have healing problems.

Panorex radiographs, computed tomography (CT), and magnetic resonance imaging (MRI) are helpful in diagnosing BRONJ. A study comparing the CT and MRI imaging findings of BRONJ with histopathology findings after resection and the extent of involved tissue showed consistency [9]. Hybrid SPECT/CT 99mTc-methylene diphosphonate 3-phase bone scintigraphy also consistently identifies BRONJ when present [10]. The characteristic CT findings of BRONJ, though nonspecific, are bony sclerosis, periosteal reaction, and bone sequestration in advanced stages, with differential diagnosis including chronic sclerosing osteomyelitis of the jaw, osteoradionecrosis, metastasis, and Paget disease [9, 11]. MRI findings include a low signal on T1-weighted, T2-weighted, and inversion recovery (IR) images suggesting low water content correlated with a paucity in cells and vessels. In contrast, the subjacent unexposed diseased bone often demonstrates T1 hypointensity and T2 and IR hyperintensity, which suggests high water content and inflammation, associated with hypercellularity, osteogenesis, and hypervascularity [11].

Radiation therapy (RT) as a cause of osteoradionecrosis (ORN) is well established. In this case, it was unlikely for the RT to have caused ORN because this is a delayed toxicity and is usually (70–94%) noted few years after completion of RT. The patient's mandibular bone lesion was present even prior to the initiation of RT. Early onset (<2 years) can be due to higher radiation doses ~70 Gy but can be accelerated due to surgical trauma [12].

American Association of Maxillofacial Surgeons (AAOMS) staging system has divided BRONJ into four stages (0–3) (Table 1) ranging from no clinical evidence of necrotic bone to exposed bone with varying degrees of symptomatology [1].

Treatment of BRONJ is complex and controversial. AAOMS has also developed treatment recommendations based on the clinical stage of BRONJ [1]. If systemic conditions permit, discontinuation of bisphosphonates may be beneficial in stabilizing BRONJ, reducing the risk of development at a new site and clinical symptoms. However, it should be noted that many bisphosphonates have long half-lives and BRONJ can be seen long after the cessation of the bisphosphonate therapy.

## 4. Conclusion

In cancer patients receiving intravenous bisphosphonate therapy, osteonecrosis of the jaw can be easily mistaken for a metastatic site due to its clinical presentation and imaging characteristics. The practicing oncologist, the diagnostic radiologist, and/or nuclear medicine specialist and the dental specialist must all be aware of BRONJ as an entity mimicking bone metastasis. Early recognition will facilitate early diagnosis, minimize the need for biopsies and multiple unnecessary imaging studies, and, most importantly, allow appropriate treatment measures to be initiated.

## Figures and Tables

**Table 1:** AAOMS staging system of BRONJ.

Stage	Clinical findings	Treatment recommendations
Stage 0	(i) Nonspecific (ii) No clinical evidence of necrotic bone	Systemic management, including pain medication and antibiotics

Stage I	Exposed, necrotic bone	(i) Antibacterial mouth rinse (ii) Follow-up on a quarterly basis patient education

Stage II	(i) Painful (ii) Exposed necrotic bone (iii) Infection of the wound cavity	(i) Oral antibiotics (ii) Oral antibacterial mouth rinse, pain control (iii) Superficial debridement to relieve soft tissue irritation

Stage III	(i) Painful (ii) Exposed, necrotic bone (iii) Wound infection (iv) Pathologic fracture/oral cutaneous fistula/osteolysis extending to the inferior border of the mandible	(i) Antibacterial mouth rinse (ii) Antibiotic therapy (iii) Pain control (iv) Surgical debridement/resection for longer-term palliation of infection and pain
